# Bio‐Inspired Microreactors Continuously Synthesize Glucose Precursor from CO_2_ with an Energy Conversion Efficiency 3.3 Times of Rice

**DOI:** 10.1002/advs.202305629

**Published:** 2023-12-03

**Authors:** Yujiao Zhu, Fengjia Xie, Tommy Ching Kit Wun, Kecheng Li, Huan Lin, Chi Chung Tsoi, Huaping Jia, Yao Chai, Qian Zhao, Benedict Tsz‐woon Lo, Shao‐Yuan Leu, Yanwei Jia, Kangning Ren, Xuming Zhang

**Affiliations:** ^1^ Department of Applied Physics The Hong Kong Polytechnic University Kowloon Hong Kong 999077 China; ^2^ Department of Chemistry Hong Kong Baptist University Kowloon Hong Kong 999077 China; ^3^ Research Centre for Resources Engineering towards Carbon Neutrality (RCRE) The Hong Kong Polytechnic University Kowloon Hong Kong 999077 China; ^4^ Department of Applied Biology and Chemical Technology The Hong Kong Polytechnic University Kowloon Hong Kong 999077 China; ^5^ Beijing Key Laboratory for Green Catalysis and Separation Department of Chemical Engineering Beijing University of Technology Beijing 100124 China; ^6^ Photonics Research Institute The Hong Kong Polytechnic University Kowloon Hong Kong 999077 China; ^7^ Department of Civil and Environmental Engineering The Hong Kong Polytechnic University Kowloon Hong Kong 999077 China; ^8^ State‐Key Laboratory of Analog and Mixed‐Signal VLSI, Institute of Microelectronics Faculty of Science and Technology – ECE Faculty of Health Sciences and MoE Frontiers Science Center for Precision Oncology University of Macau Macau 999078 China

**Keywords:** artificial photosynthesis, enzyme immobilization, layer‐by‐layer, microfluidics, microreactors

## Abstract

Excessive CO_2_ and food shortage are two grand challenges of human society. Directly converting CO_2_ into food materials can simultaneously alleviate both, like what green crops do in nature. Nevertheless, natural photosynthesis has a limited energy efficiency due to low activity and specificity of key enzyme D‐ribulose‐1,5‐bisphosphate carboxylase/oxygenase (RuBisCO). To enhance the efficiency, many prior studies focused on engineering the enzymes, but this study chooses to learn from the nature to design more efficient reactors. This work is original in mimicking the stacked structure of thylakoids in chloroplasts to immobilize RuBisCO in a microreactor using the layer‐by‐layer strategy, obtaining the continuous conversion of CO_2_ into glucose precursor at 1.9 nmol min^−1^ with enhanced activity (1.5 times), stability (≈8 times), and reusability (96% after 10 reuses) relative to the free RuBisCO. The microreactors are further scaled out from one to six in parallel and achieve the production at 15.8 nmol min^−1^ with an energy conversion efficiency of 3.3 times of rice, showing better performance of this artificial synthesis than NPS in terms of energy conversion efficiency. The exploration of the potential of mass production would benefit both food supply and carbon neutralization.

## Graphical abstract

This paper tackles carbon dioxide (CO_2_ ) and food shortage by developing a D‐ribulose‐1,5‐bisphosphate carboxylase/oxygenase (RuBisCO)‐immobilized microfluidic reactor, mimicking thylakoid's structure, to convert CO_2_ into glucose precursor. The glucose precursor production shows exceptional activity, stability, and reusability. Scaling out to multiple parallel microreactors demonstrates impressive production rates and energy efficiency, promising sustainable food supply and carbon neutralization.

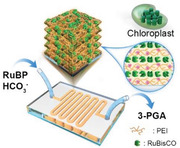

## 1 Introduction

Global carbon dioxide (CO_2_) emissions have reached a record high, triggering greenhouse effect and leading to extreme climate change, seriously damaging global food reserves and threatening human life.^[^ ^1^ ^]^ Natural green crops can simultaneously reduce atmospheric CO_2_ and supply food for human with nature photosynthesis (NPS) by directly converting CO_2_ into carbohydrates, such as glucose and starch.^[^ ^2^ ^]^ However, this conversion occurs at a very low energy efficiency (<1%),^[^ ^3^ ^]^ even after the evolution for billions of years, and the overall process is also constrained by other factors such as soil, climate, water, and labor availability. Moreover, food systems that require land clearing and deforestation, production and use of fertilizers, and combustion of fossil fuels, contribute up to one‐third of global greenhouse gas emissions, severely hindering the realization of “carbon neutrality”.^[^ ^4^ ^]^ Therefore, unprecedented changes in food production system should be integrated into climate policy. One potential technological solution is using a highly efficient system to directly convert CO_2_ into basic food materials. This could not only alleviate the food shortage problem but also contribute to carbon neutrality and ambitious goals related to green and low carbon.

Recent research on food system changes commonly focuses on improving the NPS efficiency of crops.^[^ ^5^ ^]^ In this regard, ribulose‐1,5‐bisphosphate carboxylase/oxygenase (RuBisCO, EC 4.1.1.39), the most abundant protein on Earth and the major bottleneck of NPS due to its low catalytic efficiency and poor CO_2_/O_2_ selectivity, has accordingly received considerable attention. Some C_4_ plants, algae, and cyanobacteria have better photocatalytic efficiency than the C_3_ plants by concentrating RuBisCO into intracellular compartments associated with CO_2_‐concentrating mechanisms.^[^ ^6^ ^]^ This feature inspired some studies to immobilize RuBisCO on artificial structures with enhanced selectivity, stability and reusability.^[^ ^7^ ^]^ Zhu et al. presented an advanced immobilization method for RuBisCO within microfluidic reactors that mimicked the fluidic environment of chloroplasts, demonstrating the continuous production of glucose precursor.^[^ ^8^ ^]^ Enzymes immobilized on microfluidic reactors can be easily isolated from products, therefore precluding contamination and the need for additional enzyme inactivation and separation steps.^[^ ^9^ ^]^ In addition, the unique advantages of microfluidics, such as low reagent consumption, large specific surface area, fast diffusion, and superior controllability, facilitate and expedite mechanistic studies in the fields of chemistry, chemical engineering, and biotechnology.^[^ ^10^ ^]^ The precise control of local conditions (such as temperature, pH value, reaction time, etc.) in different regions of microfluidic reactors also allows the multiple enzymatic reactions like the Calvin cycle to be cascaded and optimized,^[^ ^11^ ^]^ showing great potential for photosynthesis of glucose on a chip. Moreover, the facile scalability of microfluidic reactors presents a promising opportunity in the future for achieving continuous and large‐scale production of glucose from CO_2_,^[^ ^8b^ ^]^ contributing to food system changes related to climate policy. However, such investigations immobilized RuBisCO by means of covalent immobilization or physical adsorption, which often caused a notable degradation of its activity. And the production rates were limited due to the small surface area of microreactors for immobilizing the enzyme. It is therefore crucial to find suitable methods for RuBisCO immobilization in microfluidic reactors to preserve and increase its activity to the maximum extent. Moreover, the microfluidic reactor has a limited flow rate (e.g., 1 µL min^−1^) and needs a substantial scaling‐up process to achieve a meaningful production rate (e.g., 1 kg h^−1^). Therefore, this scaling issue is worth exploring.

**Figure 1:**
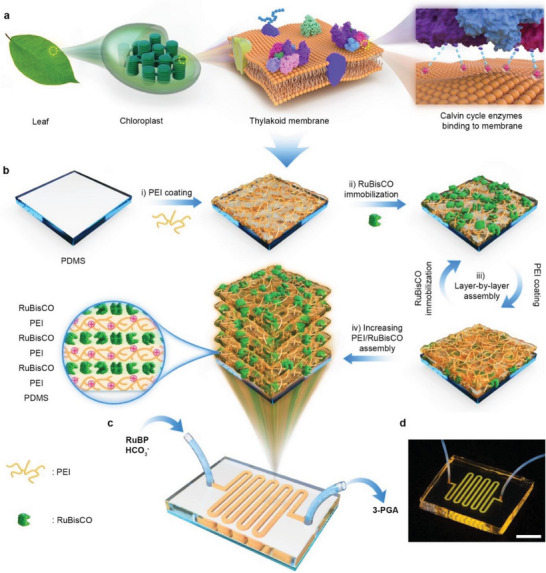
Concept and fabrication of biomimetic microfluidic reactor that immobilizes RuBisCO by layer‐by‐layer (LBL) deposition. a) In the chloroplast of a leaf, thylakoids stack together, and Calvin cycle enzymes are bound on these thylakoid membranes by electrostatic attraction indicated by the light blue dashed lines. b) A PEI layer is first coated on the sidewalls of polydimethylsiloxane (PDMS)‐based microfluidic reactor (PMR) and then utilized to adsorb RuBisCO by electrostatic attraction. The PEI coating and RuBisCO adsorption steps are then repeated several times to mimic multilayered thylakoids and to increase the RuBisCO loading amount in the PMRs. c) 3D diagram and d) photograph of fabricated (PEI/RuBisCO)*_n_*‐PMRs (*n* denotes to the number of deposition layer of PEI/RuBisCO assemblies). ribulose 1,5‐bisphosphate (RuBP) and bicarbonate (HCO_3_^−^) are injected into the inlet, and 3‐phosphoglycerate (3‐PGA) is collected from the outlet. The scale bar of the photograph is 1 cm. Only part of PEI and half of the large and small subunits of RuBisCO structures are illustrated in the schematics.

In green plant cells, NPS occurs around the thylakoid membranes in chloroplasts. It is reported that the negatively charged thylakoid membranes attract cations to maintain electroneutrality, and then the resulting cationic membrane adsorbs negatively charged Calvin cycle‐related enzymes via electrostatic attraction^[^ ^12^ ^]^ (**Figure** **1**a). Thylakoids further stack together to form grana with a large surface area to host more enzymes.^[^ ^13^ ^]^ Electrostatic attraction has been extensively utilized in scientific studies to immobilize enzymes using polyelectrolytes as the linker.^[^ ^14^ ^]^ Moreover, a layer‐by‐layer (LBL) assembly strategy, which comprises the sequential deposition of enzymes and polyelectrolytes, has usually been implemented to increase the amount of immobilized enzyme.^[^ ^15^ ^]^ Electrostatic adsorption of enzymes has been shown to promote low activity loss and to enhance stability thanks to the ideal protective microenvironment provided by the LBL assembly with mild conditions, high hydrophilicity, and excellent biocompatibility.^[^ ^16^ ^]^ Of the various polyelectrolytes available, the commonly used long‐chain branched polyethyleneimine (PEI) has abundant cationic active sites and is reported to immobilize enzymes effectively and securely.^[^ ^17^ ^]^ PEI is also a particularly attractive choice for this application as it has an outstanding CO_2_ adsorption ability and could strengthen enzymatic CO_2_ conversion.^[^ ^18^ ^]^ Despite the benefits, current scientific literature lacks research utilizing PEI and LBL assembly strategy to immobilize RuBisCO within microfluidic reactors.

This study alternately deposited branched PEI and RuBisCO on the inner walls of polydimethylsiloxane (PDMS)‐based microfluidic reactors (PMRs) to fabricate (PEI/RuBisCO)*_n_*‐PMRs (*n* denotes to the number of deposition layer of PEI/RuBisCO assemblies, the detailed fabrication process is shown in Figure 1b) with the aim to advance the objective of producing glucose with RuBisCO‐immobilized microfluidic reactors, while maximumly preserving enzymatic activity and stability. It mimics the behavior of Calvin‐cycle enzymes bound to stacked thylakoids membranes via electrostatic interactions. The RuBisCO immobilization concentration and the deposition layers of the PEI/RuBisCO assemblies were adjusted to maximize the amount of protein loaded and the RuBisCO activity. We then injected the fabricated (PEI/RuBisCO)*_n_*‐PMRs with ribulose 1,5‐bisphosphate (RuBP) and CO_2_ at different flow rates to produce the glucose precursor 3‐phosphoglycerate (3‐PGA). The (PEI/RuBisCO)*_n_*‐PMRs at optiaml conditions were found to show high catalytic efficiency in producing 3‐PGA at 1.9 nmol min^−1^, and they retained 96% activity after 10 cycles of reuse. Their thermal stability and storage stability were also confirmed. Finally, we further scaled out the (PEI/RuBisCO)*_n_*‐PMRs from one reactor into six parallel reactors and produce 3‐PGA continuously as a proof of concept for the large‐scale synthesis of 3‐PGA. The 3‐PGA production rate was improved by 8.3 times and the operation time is shortened by a factor of six. The energy conversion efficiency reached 3.3 times of rice. These findings will move one step forward to mass produce basic food materials from CO_2_, providing a technological solution to the problems of food shortage and excessive carbon emissions.

## 2 Results and Discussion

### 2.1 Confirmation of RuBisCO Immobilization

**Figure 2:**
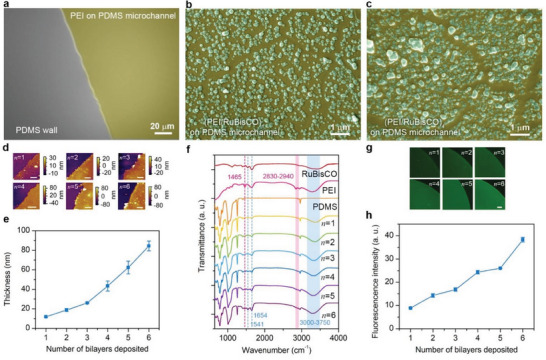
field emission scanning electron microscopy (FESEM), atomic force microscopy (AFM), attenuated total reflection‐Fourier transform infrared spectroscopy (ATR‐FTIR), and confocal laser scanning microscopy (CLSM) confirm successful RuBisCO immobilization and LBL assembly via PEI. a–c) FESEM images showing the microchannel inner surfaces of the PEI‐PMRs, where PEI is colored yellow and RuBisCO is colored green (a), (PEI/RuBisCO)_1_‐PMRs (b), and (PEI/RuBisCO)_4_‐PMRs (c). AFM images of the microchannel inner surfaces of the (PEI/RuBisCO)*_n_*‐PMRs d) and the corresponding assembled thickness as a function of the number of bilayers deposited e). In (d), the scale bars are 5 µm. In (e), the error bars represent the standard deviations of thickness values measured at three different locations of one reactor. f) ATR‐FTIR spectra of RuBisCO, PEI solution, PDMS film and the (PEI/RuBisCO)*_n_* assembly on PDMS films. g) CLSM images of the (PEI/RuBisCO)*_n_*‐PMRs and h) the corresponding fluorescence intensity as a function of the number of bilayers deposited. In (g), the scale bar is 100 µm, and in (h), the error bars represent the standard deviations of fluorescence intensities measured at three different locations of one reactor.

The 3D diagram and photograph of the fabricated (PEI/RuBisCO)*_n_*‐PMRs are shown in Figure 1c,d, respectively. To ensure the microreactor's effectiveness in 3‐PGA production, it is imperative to ensure the successful immobilization of the enzyme before any further tests. We performed several different analyses to evaluate the texture and composition of the microchannels of PMRs before and after PEI/RuBisCO assembly deposition. As observed with field emission scanning electron microscopy (FESEM) (**Figure** **2**a–c), the pristine PDMS sidewall before the PEI coating was smooth and flat, and no obvious change in texture was observed after the PEI coating was applied (Figure 2a). The inner surfaces of the (PEI/RuBisCO)_1_‐PMR microchannels consisted of a PEI layer decorated with small particles after RuBisCO deposition (Figure 2b), implying the successful immobilization of RuBisCO by PEI. A greater density of RuBisCO particles and an obvious layer of mixed RuBisCO and PEI were observed after the subsequent deposition of a PEI/RuBisCO bilayer (Figure S3b, Supporting Information). As the number of PEI/RuBisCO bilayers increased, the distribution of the RuBisCO particles became denser, and small particles tended to aggregate into larger particles (Figure 2c; Figure S3, Supporting Information). A similar morphology with the mixed layer of RuBisCO particles and PEI was also observed. The FESEM results were limited to the surface morphology of the inner surfaces of microreactors. To gain a more comprehensive understanding of the multilayer structure of the deposited layers, atomic force microscopy (AFM) measurements were further conducted to examine the 3D topography in greater detail. The inner surfaces of the (PEI/RuBisCO)*_n_*‐PMR microchannels showed a thickening of the PEI/RuBisCO assembly as the number of bilayers increased (Figure 2d). The average thickness of the (PEI/RuBisCO)*_n_* assembly increased from 11.8 to 84.5 nm for *n* = 1 to 6 (Figure 2e), indicating that more PEI/RuBisCO was deposited, as expected. Here, FESEM and AFM results confirm the successful LBL deposition of PEI/RuBisCO by presenting the topography of the inner surfaces of microchannels.

Whilst FESEM and AFM served to elucidate the topography of the deposited layers, we complemented these techniques by further employing attenuated total reflectance‐Fourier transform infrared (ATR‐FTIR) spectroscopy and circular dichroism (CD) spectroscopy to interrogate the molecular structure of the inner surfaces of the microchannels before and after enzyme immobilization. As shown by the ATR‐FTIR spectra in Figure 2f, the spectra obtained with the (PEI/RuBisCO)_1_ assembly coating exhibited new peaks at 1400−1800 cm^−1^, 2800–2900 cm^−1^, and 3000−3750 cm^−1^ compared with the spectra of the pristine PDMS film, which were assigned to C‐H/N‐H bending, C‐H stretching and N‐H stretching vibrations, respectively. Moreover, the peaks at ≈1500−1600 cm^−1^ and 1600−1700 cm^−1^ were assigned to the vibrational modes of the amide II and amide I bands, specifically indicating the secondary structure of the assembled RuBisCO. Note that the intensity of the peak at 1500−1700 cm^−1^ increased with larger *n* values, supporting the previous FESEM and AFM observations that more RuBisCO was deposited with increasing *n*. These transmittance bands confirmed the presence of the PEI coating and the effective RuBisCO immobilization on PEI‐coated PDMS. Similar results were also obtained by measuring the CD spectra of the (PEI/RuBisCO)*_n_* assembly on PDMS films to confirm RuBisCO immobilization (Figure S4, Supporting Information).

To confirm the augmentation of immobilized RuBisCO amount following LBL assembly, we labeled the immobilized RuBisCO with primary anti‐RuBisCO and Alexa Fluor 488‐conjugated goat anti‐chicken IgG (H+L) secondary antibody (AF488 GTXCH). As shown by the confocal laser scanning microscopy (CLSM) images in Figure 2g, the microchannels of all the (PEI/RuBisCO)*_n_*‐PMRs clearly showed green fluorescence, and the fluorescence intensity increased as *n* increased from 1 to 6 (Figure 2h). This again verifies the immobilization of more RuBisCO in the (PEI/RuBisCO)*_n_*‐PMRs. Overall, the results of FESEM, AFM, ATR‐FTIR, CD and CLSM analyses consistently confirmed the successful immobilization of RuBisCO in microreactor, and the immobilization amount increases with the deposition layer.

### 2.2 Optimization of (PEI/RuBisCO)*_n_*‐PMRs Fabrication

**Figure 3:**
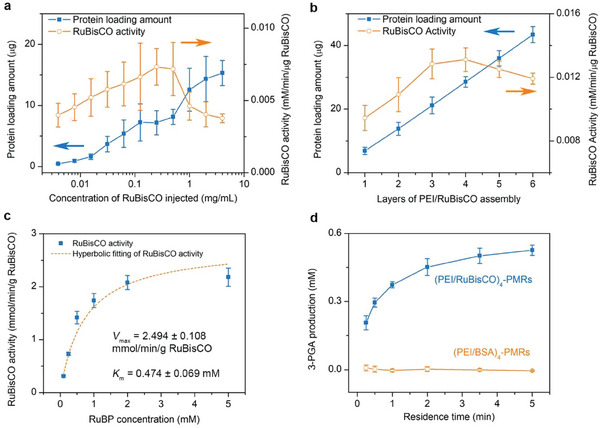
Optimization of the fabrication conditions for (PEI/RuBisCO)*_n_*‐PMRs and their tested performance. a) The protein‐loading amount and immobilized RuBisCO activity of the (PEI/RuBisCO)_1_‐PMRs fabricated with increasing concentrations of RuBisCO injected from 0.0039 to 4 mg mL^−1^. Solutions of 0.5 mm RuBP and 66 mm HCO_3_^−^ were injected at a flow rate of 7 µL min^−1^ for the activity determination. The relative activity peaks at 0.25 mg mL^−1^. b) The protein‐loading amount and immobilized RuBisCO activity of the (PEI/RuBisCO)*_n_*‐PMRs as a function of the increasing number of PEI/RuBisCO assembly layers. The relative activity peaks at *n* = 4. Solutions of 0.5 mM RuBP and 66 mM HCO_3_^−^ were injected at a flow rate of 7 µL min^−1^ for the activity determination. According to (a) and (b), the optimal conditions are chosen to be a RuBisCO concentration of 0.25 mg mL^−1^ and 4 layers for the PEI/RuBisCO assembly. The orange and blue arrows in (a) and (b) serve to indicate that the two different‐colored curves correspond to different *y*‐axis scales. c) The kinetic parameters *K*_m_ and *V*_max_ of the (PEI/RuBisCO)_4_‐PMRs were calculated by hyperbolic regression fitting of the Michaelis–Menten model using GraphPad Prism 7. The volume of product solution collected was 100 µL. The RuBP concentrations are 0.125–5 mm for the (PEI/RuBisCO)_4_‐PMR reaction. The concentration of HCO_3_^−^ in the reaction buffer was 66 mm. d) 3‐PGA production as a function of the residence time using (PEI/RuBisCO)_4_‐PMRs and (PEI/BSA)_4_‐PMRs (PEI/bovine serum assembly immobilized onto the PMRs in four layers) as the control. Solutions of 0.5 mm RuBP and 66 mm HCO_3_^−^ were injected at different flow rates to control the residence time. Error bars represent the standard deviations from three independent experiments.

Having confirmed the successful immobilization of RuBisCO, we next turned to optimize the conditions for (PEI/RuBisCO)*_n_*‐PMRs fabrication in order to maximize 3‐PGA production. By evaluating the protein‐loading amount and the immobilized RuBisCO activity per microreactor, the concentration of RuBisCO in the (PEI/RuBisCO)_1_‐PMRs was first optimized and then used to optimize the deposition layer of PEI/RuBisCO assembly in the (PEI/RuBisCO)*_n_*‐PMRs. As shown in **Figure** **3**a, with increasing RuBisCO concentration from 0.0039 to 4 mg mL^−1^, the protein‐loading amount of a single (PEI/RuBisCO)_1_‐PMR increased from 0.45 to 15.30 µg (i.e., more RuBisCO was immobilized in the reactors). However, the activity of the immobilized RuBisCO reached saturation at 0.0075 mm min^−1^ µg^−1^ RuBisCO at a RuBisCO concentration of 0.25 mg mL^−1^. This phenomenon was ascribed to steric hindrance that restrained the diffusion of the substrates and products to the immobilized enzyme layer after excessive and agglomerated enzyme loading on the inner surface of the microreactors.^[^ ^19^ ^]^ Therefore, a RuBisCO concentration of 0.25 mg mL^−1^ was chosen as the working condition for the subsequent fabrication of multilayer (PEI/RuBisCO)*_n_*‐PMRs.

(PEI/RuBisCO)*_n_*‐PMRs were fabricated by repeating the deposition of PEI/RuBisCO bilayers in the PMRs for *n* times. The protein‐loading amounts increased almost linearly from *n* = 1 to 6 (blue squares in Figure 3b), indicating that the PEI layer offers an ideal matrix for RuBisCO immobilization even in a repeated deposition process. The overall catalytic activity improved with an increasing number of PEI/RuBisCO bilayers due to the increase in the protein‐loading amount, but the RuBisCO activity peaked at 0.013 mm min^−1^ µg^−1^ RuBisCO with *n* = 4 (brown circles in Figure 3b), potentially due to the greater diffusion distance between the reactant and the immobilized RuBisCO in the inner layers for PMRs with *n* > 4.^[^ ^6^, ^20^ ^]^ Therefore, *n* = 4 was considered the optimal layer number for further investigation and the protein‐loading amount of one (PEI/RuBisCO)_4_‐PMR was 28.5 µg.

### 2.3 Kinetics Study of RuBisCO Immobilized by LBL Assembly

After the optimal conditions were determined for (PEI/RuBisCO)*_n_*‐PMRs fabrication (initially injected RuBisCO concentration of 0.25 mg mL^−1^ and *n* = 4), the evaluation of kinetic parameters is crucial for characterizing the affinity of the immobilized RuBisCO for the substrate and the maximal reaction rate, thereby enabling the optimization of substrate concentration for achieving maximum reaction rates and efficiency. Before the kinetics study, sodium dodecyl sulfate–polyacrylamide gel electrophoresis (SDS‐PAGE) analysis was conducted to estimate the relative purity of the purchased RuBisCO as shown in Figure S7a (Supporting Information). Then, the kinetic parameters, including Michaelis–Menten constant (*K*_m_), implying the affinity of enzyme for the substrate, maximal reaction rate (*V*_max_), turnover number (*k*_cat_), and catalytic efficiency (*k*_cat_/*K*_m_) were estimated for (PEI/RuBisCO)_4_‐PMRs and (PEI/RuBisCO)_1_‐PMRs. This was accomplished by experimentally evaluating the activity of RuBisCO immobilized by LBL assembly with varying concentrations of RuBP substrate and comparing the results with free RuBisCO and other immobilization methods in the previous studies.^[^ ^8^ ^]^ For the case of *n* = 1, the (PEI/RuBisCO)_1_ assembly had a *K*_m_ value of 0.378 mm and a *V*_max_ value of 1.146 mmol min^−1^ g^−1^ RuBisCO, which were higher than those of free RuBisCO (i.e., 0.049 mm and 0.169 mmol min^−1^ g^−1^ RuBisCO, Figure S7 and Table S1, Supporting Information). This higher *K*_m_ value implied a reduced affinity of the immobilized RuBisCO for the substrate, which may be due to the increased diffusion distance and the steric hindrance of some active sites after immobilization. For the case of *n* = 4, the *K*_m_ value for the (PEI/RuBisCO)_4_ assembly was even larger (Figure 3c), implying a greater diffusion limitation for multilayer deposition. Nevertheless, the *V*_max_ value of (PEI/RuBisCO)*_n_* was enhanced by 1.4‐fold for *n* = 1 and 14.8‐fold for *n* = 4 as compared to free RuBisCO; the turnover number *k*_cat_ and catalytic efficiency *k*_cat_/*K*_m_ were also improved significantly by 15.1‐fold and1.6‐fold for *n* = 4, respectively (Table S1, Supporting Information). Consistent with the results of the preceding section, the increased number of immobilization layers allowed for a higher concentration of enzyme to be present in the microreactor, leading to a greater number of enzyme‐substrate interactions and higher reaction rates. The increased reaction rates may also be attributed to the specific surface area and high mass transfer efficiency offered by the microfluidic reactors. However, the multilayered immobilized enzyme also led to a greater diffusion distance, which subsequently increased the *K*_m_ value. Nevertheless, the obtained *K*_m_ value enabled the determination of the optimal substrate concentration for the following 3‐PGA production, namely 0.5 mm of RuBP.

Subsequently, we compared *V*_max_ for the LBL‐assembled RuBisCO with those obtained for covalently immobilized and physically adsorbed RuBisCO in the previous studies (Table S1, Supporting Information). The results showed an increase in *V*_max_ for the LBL‐assembled RuBisCO, indicating a significant improvement of the enzyme activity by LBL deposition. We next analyzed the charge distribution of RuBisCO with the aim of better understanding these effects. According to Figure S8a–c (Supporting Information), there are a large number of negatively charged sites on the surface of RuBisCO for electrostatic interaction with PEI, and positively charged sites dominate the regions near the active center. This implies that there are few opportunities for active centers to interact with PEI, thus precluding an effect on RuBisCO activity. However, for physical adsorption, as presented in a previous work,^[^ ^8b^ ^]^ hydrophobic sites prevail near the active center (Figure S8d, Supporting Information), which may greatly affect the exposure of active sites and RuBisCO activity. For covalent immobilization,^[^ ^8a^ ^]^ the amino groups of lysine residues at the active sites of RuBisCO are of crucial importance in catalysis (Figure S8e, Supporting Information), potentially causing irreversible conformational changes and activity reduction after covalent immobilization.^[^ ^21^ ^]^ Therefore, electrostatic attraction and LBL assembly have less effect on RuBisCO activity than covalent immobilization and physical adsorption, resulting in the larger *V*_max_ of the (PEI/RuBisCO)_4_‐PMRs. The analysis is also coherent with previous reports that the random coil structure of PEI does not allow much distortion of the enzyme after its association through multiple points^[^ ^16c^ ^]^ and PEI layers offer an ideal biocompatible microenvironment for enzyme immobilization.^[^ ^16a^ ^]^ The exceptional CO_2_ adsorption capabilities of PEI may also contribute to the enhancement in the catalytic efficiency,^[^ ^18^ ^]^ which are not accessible in the cases of covalent immobilization and physical adsorption.

### 2.4 Feasibility of 3‐PGA Production in (PEI/RuBisCO)*_n_*‐PMRs with Optimal Assembled Layers

After the preparation of the (PEI/RuBisCO)_4_‐PMRs under the optimal deposition conditions, the feasibility of 3‐PGA production was studied by injecting RuBP with the optimal concentration of 0.5 mm and 66 mm HCO_3_^−^ at different flow rates to control the residence time. The amount of produced 3‐PGA increased with the prolonged residence time (Figure 3d) and tended to saturate when the residence time was > 2 min, which may be attributed to the depletion of RuBP, as explained by previous studies.^[^ ^8^, ^22^ ^]^ The yield of 3‐PGA reached 52.7% at 5 min. In the control experiment, bovine serum albumin (BSA) was immobilized onto the microfluidic reactors (referred to as (PEI/BSA)_4_‐PMRs), and 3‐PGA production could not be triggered (Figure 3d). This result proved that the glucose precursor 3‐PGA could be successfully synthesized through the Calvin cycle pathway on the (PEI/RuBisCO)_4_‐PMRs with high efficiency.

### 2.5 Thermal and Storage Stability of (PEI/RuBisCO)*_n_*‐PMRs with Optimal Assembled Layers

**Figure 4:**
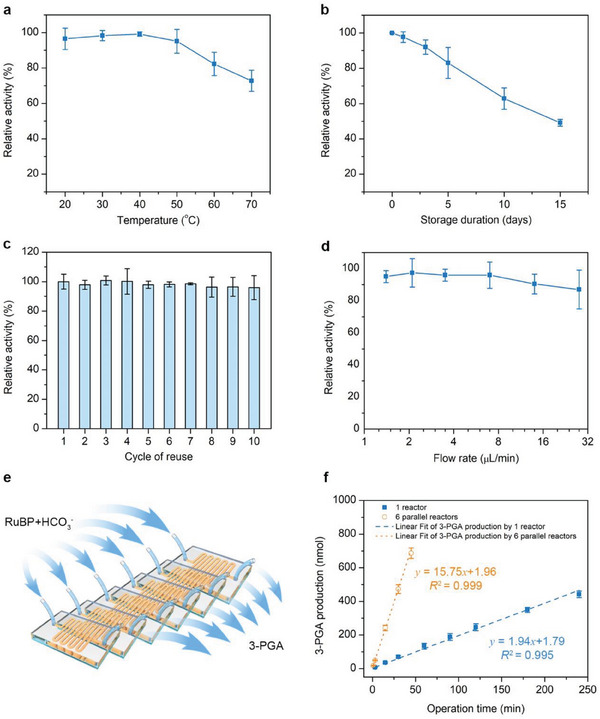
Stability, reusability and scaling out of the (PEI/RuBisCO)_4_‐PMRs conceptually demonstrate their potential in large‐scale production. a) The thermal stability of the (PEI/RuBisCO)_4_‐PMRs showing the relative activities of immobilized RuBisCO after incubation at different temperatures from 20 to 70 °C. All samples are incubated for 10 min before the activity assay. b) The storage stability of the immobilized RuBisCO with each sample incubated at 4°C for up to 15 days. c) Reusability tests of the (PEI/RuBisCO)_4_‐PMRs showing the retained relative activities after 10 cycles of reuse at the flow rate of 7 µL min^−1^. d) The relative activity retained after 10 cycles of reuse varies with the flow rate of the injected RuBP and HCO_3_^−^ (from 28 to 1.4 µL min^−1^). The *x*‐axis is in the log2 scale. e) Diagram of scaling out into 6 reactors. f) The continuous production of 3‐PGA shows that the amount increases linearly with the total operation time. The (PEI/RuBisCO)_4_‐PMRs were prepared with 0.25 µg µL^−1^ RuBisCO and an immobilization time of 4 h. RuBP (0.5 mm) and HCO_3_^−^ (66 mm) were injected at a flow rate of 7 µL min^−1^ unless otherwise stated. Error bars represent the standard deviations from three independent experiments.

Thermal and storage stability of the enzyme is of great importance for the operation of the (PEI/RuBisCO)_4_‐PMRs in future industrial application. Therefore, we next evaluated the activity of (PEI/RuBisCO)_4_‐PMRs at an elevated temperature (from 20 to 70 °C) and with a prolonged storage time (up to 15 days). The highest activity (*A*_ht_) of the (PEI/RuBisCO)_4_‐PMRs appeared at 40 °C (**Figure** **4**a). In the range of 20–40 °C, a temperature increase resulted in more activated molecules to accelerate the reaction rate. At temperatures higher than 40 °C, the apparent activity decreased, likely due to the structure changes of the enzyme at elevated temperature.^[^ ^19b^ ^]^ The optimal incubation temperature range (i.e., over 98% of *A*_ht_ retained after incubation) of RuBisCO shifted from the range of 24–34 °C for free RuBisCO (a temperature range Δ*T* = 10°C, as determined in the previous work^[^ ^8a^ ^]^) to 28–45 °C after the LBL assembly (Δ*T* = 17 °C). Compared with the ranges obtained in previous studies for covalently immobilized (29–40 °C, Δ*T* = 11°C)^[^ ^8a^ ^]^ and physically adsorbed RuBisCO (33–42 °C, Δ*T* = 9°C),^[^ ^8b^ ^]^ the temperature range Δ*T* was obviously expanded for the enzyme after LBL assembly via electrostatic attraction (17°C vs ≈10°C).

At an elevated temperature of 70 °C, the retained activity of the immobilized RuBisCO was approximately 72% *A*_ht_, which was 7.2 times the retained activity of 10% *A*_ht_ at 70 °C for free RuBisCO,^[^ ^8a^ ^]^ 1.07 times that of covalently immobilized RuBisCO (67%)^[^ ^8a^ ^]^ and 1.26 times that of physically adsorbed RuBisCO (57%)^[^ ^8b^ ^]^ (Table S2, Supporting Information). When incubated at 50 °C for a prolonged time, up to 60 min, the LBL‐assembled RuBisCO retained 74% *A*_ht_, very close to that of covalently immobilized RuBisCO (75%)^[^ ^8a^ ^]^ and higher than those of physically adsorbed RuBisCO (65%)^[^ ^8b^ ^]^ and free RuBisCO (46%)^[^ ^8a^ ^]^ (Figure S9, Supporting Information). Electrostatic attraction resulted in a slightly enhanced thermal stability compared to covalent immobilization (i.e., 72% vs 67%). However, these results showed that both electrostatic attraction and covalent immobilization significantly enhance the resistance of RuBisCO to thermal inactivation at an elevated temperature compared to free enzyme or physical adsorption.

Afterwards, we evaluated the storage stability by measuring the enzyme half‐life (the duration after which 50% of the initial activity is retained) after storage in reaction buffer at 4 °C. The (PEI/RuBisCO)_4_‐PMRs exhibited a half‐life of ≈14 days (Figure 4b), which was 5.4 times that of the free RuBisCO (2.6 days) and 1.8 times that of covalently immobilized RuBisCO (8 days).^[^ ^8a^ ^]^ In addition, after incubation at 4 °C for 15 days, the storage stability of the RuBisCO immobilized in the (PEI/RuBisCO)_4_‐PMRs (with 49% initial activity retention) was 8.2 times greater than that of free RuBisCO (6%) and 1.1 times greater than that of covalently immobilized RuBisCO (43%).^[^ ^8a^ ^]^ The electrostatic interaction by LBL assembly markedly improved the stability of RuBisCO for long‐term storage. For direct comparison, Table S2 (Supporting Information) lists the major performance metrics of free RuBisCO and RuBisCO immobilized by different methods.

Both the improved thermal and storage stabilities of RuBisCO could be ascribed to the enhanced stability of the enzyme conformation after electrostatic attraction. The PEI layers of the LBL assembly offered an ideal matrix with multiple anchors on the support to fix the enzyme, which was greatly beneficial for preserving the enzyme structure and active sites. In contrast, the free enzyme, physically adsorbed enzyme, and covalently immobilized enzyme tended to undergo unfolding and structural changes due to the lack of a fixation matrix. As a result, the (PEI/RuBisCO)_4_‐PMRs were superior in maintaining enzyme activity.

### 2.6 Reusability of (PEI/RuBisCO)*_n_*‐PMRs with Optimal Assembled Layers

Reusability is another important criterion for practical applications. We refer one cycle of reuse to the test that 21 µL of reactant mixture solution passes through the microreactor for 3‐PGA production. After 10 cycles of reuse with an injection flow rate of 7 µL min^−1^ and a residence time of 1 min, the (PEI/RuBisCO)_4_‐PMRs retained approximately 96% of their initial activity (Figure 4c). By linearly fitting the relative activities after each cycle of reuse, the post‐immobilization leakage rate for (PEI/RuBisCO)_4_‐PMRs was determined to be 0.15% per cycle of reuse. The little activity loss may be due to enzyme deactivation and detachment after repeated uses and flushing with the running reactant mixture.^[^ ^19a^ ^]^

Compared with the reusability of physically adsorbed RuBisCO (40% activity retained after 10 cycles of reuse and 6.61% of post‐immobilization leakage rate)^[^ ^8b^ ^]^ and covalently immobilized RuBisCO (74% activity retained after 10 cycles of reuse and 2.94% of post‐immobilization leakage rate),^[^ ^8a^ ^]^ the reusability of LBL‐assembled RuBisCO was noticeably higher, as summarized in Table S2 (Supporting Information). We speculated that this could be ascribed to the difference between the 3D structure of the assembled PEI multilayer structure versus the 2D structure formed by physical adsorption or covalent immobilization; the former provides a very stable and protective microenvironment for the enzyme to avoid detachment induced by the shear forces between the fluid and the microchannel surfaces. It has also been reported that the oxidation of protein may be effectively prevented in multilayer assembly.^[^ ^23^ ^]^ To test this hypothesis, we additionally examined the reusability of (PEI/RuBisCO)_1_‐PMRs, which have a 2D structure similar to that produced by physical adsorption or covalent immobilization. The results showed that the relative activity retained after 10 cycles of reuse and the post‐immobilization leakage rate of the (PEI/RuBisCO)_4_‐PMRs were 1.7 times and 0.04 times that of the (PEI/RuBisCO)_1_‐PMRs, respectively (96% vs 55.4% for retained activity and 0.15% vs 4.25% for leakage rate, Figure S10, Supporting Information). This well confirmed that the 3D structure of LBL‐assembled RuBisCO was the dominant factor in reusability improvement.

Enzyme detachment due to the flushing of the running reactant mixture could also be observed by measuring the reusability at varying flow rates. When the injection flow rate of RuBP and HCO_3_^−^ was increased from 7 to 28 µL min^−1^, the activity remaining after 10 cycles of reuse was reduced from 96% to 87% as a result of the increased shear force (Figure 4d). Generally, a slower injection flow rate is associated with an increase in remaining activity, but too long of an operation time may also cause some activity loss. For example, at a flow rate of 1.4 µL min^−1^, 95% of the initial activity was retained, but at a flow rate of 2 µL min^−1^, 97% of the initial activity was retained, the highest retention rate observed. This experiment demonstrated the ease of RuBisCO reuse in the (PEI/RuBisCO)_4_‐PMRs by means of simultaneously pumping out the product solution and injecting a new reactant mixture. Moreover, this excellent reusability indicates the feasibility of using (PEI/RuBisCO)_4_‐PMRs in future large‐scale applications.

### 2.7 Continuous 3‐PGA Production by Optimized (PEI/RuBisCO)4‐PMRs

The ultimate goal of this work is to produce the glucose precursor with the fabricated (PEI/RuBisCO)_4_‐PMRs on a continuous basis and in large quantities; therefore, we constantly injected the reactant mixture into the (PEI/RuBisCO)_4_‐PMRs to produce 3‐PGA continuously. The individual (PEI/RuBisCO)_4_‐PMRs produced ≈442.2 nmol of 3‐PGA with a total operation time of 240 min (80 cycles) to collect 1680 µL of product solution (Figure 4f). The 3‐PGA production increased linearly with the operation time, and the fitted production rate was 1.9 nmol min^−1^ (*R* ^2^ = 0.995). This linearity could be ascribed to the outstanding stability of RuBisCO under repeated use and prolonged operation time.

### 2.8 Scaling‐Out of Microreactors

As a proof of concept of large‐scale production, the (PEI/RuBisCO)_4_‐PMRs were then scaled out from one reactor to six parallel reactors (Figure 4e). After scaling out, the 3‐PGA production amount increased notably, to 687.4 nmol within only 45 min (15 cycles for each microreactor) with the collection of 1890 µL of product solution (hollow circles in Figure 4f). The fitted reaction rate improved to 15.8 nmol min^−1^, 8.3 times that of a single reactor. The linearity of the production was even better (*R* ^2^ = 0.999 vs 0.995 for a single reactor). By scaling out the number of (PEI/RuBisCO)_4_‐PMRs from one to six, the operation time is shortened by a factor of six, reducing the cycles of reuse for each microreactor and thus effectively preserving the reactor's activity, so that the fitted reaction rate is 8.3 times that of a single reactor instead of six times.

It was reported that the yield of Early Rice in China was 5914.3 kg ha^−1^ in 2022^[^ ^24^ ^]^ and every 100 g of rice contains 130 kcal of energy^[^ ^25^ ^]^ (i.e., 544 kJ). Then, if the rice matures in 120 days, the energy conversion efficiency of rice would be approximately 1.86 × 10^−2^ kJ m^−2^ min^−1^, here the energy conversion efficiency refers to the energy value in the unit of kJ contained in the rice produced per minute per square meter of land. If it is assumed that 1g of carbohydrates provide 4 kcal of energy^[^ ^26^ ^]^ (i.e., 16.7 kJ), then six paralleled (PEI/RuBisCO)_4_‐PMRs achieved 4.94 × 10^−5^ kJ min^−1^. With one reactor channel area of ≈133.80 mm^2^, the energy conversion efficiency of six microreactors would be 6.13 × 10^−2^ kJ m^−2^ min^−1^, here the energy conversion efficiency refers to the energy value in the unit of kJ contained in the carbohydrates produced per minute per square meter of microchannels. This is ≈3.3 times of that of the energy of rice, showing our artificial synthesis performed better than NPS in terms of energy conversion efficiency.

Parallelization or scaling out of the (PEI/RuBisCO)_4_‐PMRs saved considerable operation time and makes the over‐repeated use of a single reactor unnecessary, proving the feasibility of large‐scale production by using the (PEI/RuBisCO)_4_‐PMRs. In theory, dozens or hundreds of (PEI/RuBisCO)_4_‐PMRs can be paralleled, and the characteristic dimensions of the channel could be easily scaled up to realize industrial‐scale 3‐PGA production in future applications. Such an integrated system would facilitate large‐scale and rapid 3‐PGA synthesis and promote the future synthesis of basic food materials from CO_2_.

## 3 Conclusion

In this work, RuBisCO was immobilized on the walls of PMR microchannels with the polycation PEI using an LBL assembly strategy to continuously produce the glucose precursor 3‐PGA in large quantities. PEI immobilized RuBisCO via electrostatic interaction, which imitated the enzyme attachment to thylakoid membranes in natural photosynthesis. The (PEI/RuBisCO)*_n_*‐PMRs showed a higher protein‐loading capacity with an increasing number of PEI/RuBisCO bilayers, and the largest activity retention was observed at *n* = 4. The (PEI/RuBisCO)_4_ multilayer presented a catalytic efficiency of 1.5‐, 5.3‐, and 60.1‐fold with respect to those of free RuBisCO, covalently immobilized RuBisCO and physically adsorbed RuBisCO, respectively. The thermal stability, storage stability and reusability were also significantly improved; RuBisCO immobilized by LBL assembly had a 7.2‐fold higher thermal stability and an 8.2‐fold higher storage stability than free RuBisCO, indicating the good protection of RuBisCO from the microenvironment offered by this biocompatible platform. The yield of 3‐PGA using the (PEI/RuBisCO)_4_‐PMRs reached 52.7% in 5 min, and ≈96% of the initial activity was retained after 10 cycles of reuse. The constant injection of CO_2_ and RuBP through the (PEI/RuBisCO)_4_‐PMRs achieved continuous and accumulative 3‐PGA production with a very small amount of RuBisCO. Scaling out from one reactor to six parallel reactors resulted in an 8.3‐fold increase in the reaction rate and a 3.3‐fold increase in energy conversion efficiency with respect to the natural rice, further demonstrating the feasibility of large‐scale synthesis using this platform.

PEI multilayers are generated here by a simple LBL strategy to provide an ideal biocompatible 3D matrix for RuBisCO immobilization via electrostatic interactions, greatly preserving the conformational structure and catalytic activity of this enzyme. Our study contributes to the expanding literature on the efficacy of the LBL strategy employing PEI for RuBisCO immobilization, and further demonstrates its superiority over covalent immobilization and physical adsorption in enhancing the catalytic efficiency of RuBisCO. While the CO_2_ capture ability of PEI has been proposed to facilitate enzymatic CO_2_ conversion, the underlying mechanism needs further investigation. Besides, the molecular weight and concentration of PEI may have a synergistic effect on enzyme immobilization, necessitating further optimization to refine this methodology. Additionally, to achieve multi‐enzymatic Calvin cycle reaction for glucose synthesis in microfluidic reactors, the applicability of this LBL strategy with PEI needs to be expanded from single‐enzyme immobilization to multi‐enzyme immobilization. Moreover, given that PEI has been reported to immobilize electron mediators and photocatalysts to integrate photocatalytic reactions with enzymatic CO_2_ conversion,^[^ ^27^ ^]^ we propose that an integrated microreactor utilizing PEI as the sole linker to immobilize photocatalysts, mediators, and enzymes simultaneously could achieve the ultimate goal of artificially photosynthesizing glucose on a chip.

Furthermore, the microfluidic technology used in our study provides several benefits, including a large specific surface area and a fast mass transfer, which result in enhanced catalytic efficiency, stability, and reusability of RuBisCO. With the scalability of microfluidic reactors, large‐scale and rapid synthesis of 3‐PGA can be achieved. However, further work is needed to address the remaining barriers to scaling up and scaling out the microreactors for practical applications. If these challenges can be overcome, integrated microfluidic reactor systems could be used to mass‐produce basic food materials (e.g., glucose) directly from CO_2_ by artificial photosynthesis, providing a technological solution to both food crisis and carbon neutralization.

## 4 Experimental Section

### Chemicals and Reagents

The reaction buffer (pH 8.0) used for the enzyme assay consisted of 0.1 m tris(hydroxymethyl)aminomethane buffer (Tris‐HCl, pH 8.0, Beijing Solarbio Technology Co., Ltd., Beijing), 5 mm magnesium chloride hexahydrate (MgCl_2_×6H_2_O, AR, Sinopharm Chemical Reagent Co., Ltd., Shanghai), 66 mm potassium bicarbonate (KHCO_3_, AR, Sinopharm Chemical Reagent Co., Ltd., Shanghai), and 5 mm DL‐dithiothreitol (DTT, 99%, Aladdin Industrial Corporation, Shanghai). Reduced nicotinamide adenine dinucleotide disodium salt (NADH Na_2_, ≥98.0%) and adenosine‐5′‐triphosphate disodium salt trihydrate (ATP×Na_2_, ≥98.0%) were purchased from Beijing Solarbio Technology Co., Ltd. Albumin (98%, from bovine serum) was purchased from J&K Scientific Ltd., Beijing. Alexa Fluor 488‐conjugated goat anti‐chicken IgG (H+L) secondary antibody (AF488 GTXCH) was purchased from Thermo Fisher Scientific. Other major reagents were provided by Sigma‒Aldrich, including polyethyleneimine solution (PEI, branched, average M_w_∼750000 by LS, 50% in H_2_O), D‐ribulose 1,5‐bisphosphate sodium salt hydrate (RuBP, ≈90%), D‐ribulose 1,5‐diphosphate carboxylase (RuBisCO, from spinach partially purified powder, 0.01–0.1 unit per mg solid), anti‐RuBisCO (plant) antibody produced in chicken, D‐(−)−3‐phosphoglyceric acid disodium salt (3‐PGA, ≥93%), glyceraldehyde 3‐phosphate dehydrogenase (GAPDH, from rabbit muscle lyophilized powder), 3‐phosphoglyceric phosphokinase (PGK, from baker's yeast (*S. cerevisiae*), ammonium sulfate suspension, ≥1000 units per mg protein), glycerol 3‐phosphate oxidase (G3POX, from *Pediococcus* sp. lyophilized powder), α‐glycerophosphate dehydrogenase (G3PDH, from rabbit muscle, type I, ammonium sulfate suspension), triosephosphate isomerase (TPI, from baker's yeast (*S. cerevisiae*), ammonium sulfate suspension) and catalase (from bovine liver powder).

### Immobilization of RuBisCO with PEI in PMRs

The PMRs were fabricated by sealing one molded PDMS layer against another flat PDMS layer on top (Figure S1, Supporting Information). The height of the microchannels is 40 µm and the detailed dimensional information of the microchannels is labeled in Figure S2 (Supporting Information). The molded layer was produced by using a standard soft photolithography technique^[^ ^28^ ^]^ with the Sylgard 184 elastomer kit (Dow Corning) and the SU‐8 mold (SU‐8 50, MicroChem). Briefly, a 4″ silicon wafer was first cleaned by acetone, ethanol, and deionized water (DIW), followed by drying in an oven to remove any contamination. Then, the SU‐8 was spin‐coated to a thickness of 40 µm (at 500 rpm for 15 s followed by 2500 rpm for 60 s) on the cleaned wafer and then soft‐baked at 65 °C for 5 min and another baking step at 95 °C for 15 min. The soft‐baked SU‐8 was then exposed to UV light for 45 s (with exposure energy of 180 mJ cm^−2^), followed by a post‐baking process at 65 °C for 5 min and at 95 °C for 15 min (as shown in Step 1 of Figure S1, Supporting Information). Afterward, the SU‐8 was developed in SU‐8 developer for 3 min and then washed with isopropyl alcohol (IPA) for 10 seconds, followed by air dry using compressed nitrogen. Finally, the mold was hard baked at 150 °C for 5 min. The SU‐8 mold was ready for further use (Step 2). Next, the PDMS compound which was formed by mixing PDMS and its curing agent at a weight ratio of 10:1 was poured onto the SU‐8 mold and put on a hot plate at 85 °C for 30 min (Step 3). The bonding of the microfluidic chips was realized by gently placing one cured molded PDMS onto another half‐cured flat PDMS slice which was baked on the hot plate for 7 min with PDMS compound poured onto a flat silicon wafer (Step 4). The two slices were finally sealed together after another 30 min of baking (Step 5).

After the fabrication of PMRs, PEI (10 mg mL^−1^) in Tris‐HCl buffer (10 mm, pH 8.8, Beijing Solarbio Technology Co. Ltd.) was injected into the PMRs by pipet and then incubated at room temperature for 1 h (Figure 1b, step i). Then, the PMRs were washed with the Tris‐HCl buffer (200 µL, 10 mm, pH 8.8) at 7 µL min^−1^ to remove any weakly absorbed PEI. Then, reaction buffer containing the indicated concentration of RuBisCO (0.00391 to 4 µg µL^−1^) was injected into the PEI‐coated PMRs at a flow rate of 2.5 µL min^−1^ for 1 h (Figure 1b, step ii). After that, reaction buffer (100 µL) was applied at the same flow rate to rinse the microreactors. In this way, RuBisCO was immobilized on the PDMS surface by electrostatic attraction, and the (PEI/RuBisCO)_1_‐PMRs were ready for use.

### RuBisCO Immobilization by Layer‐By‐Layer Assembly

Here, a single PEI layer with a RuBisCO layer on top is defined as the first PEI/RuBisCO layer. Through electrostatic interactions, another positively charged PEI layer could be coated onto the previously immobilized negatively charged RuBisCO layer, allowing for the immobilization of subsequent layer of RuBisCO. By repeating the PEI coating and RuBisCO immobilization procedures with the same conditions as the first PEI/RuBisCO layer, a PEI/RuBisCO multilayers assembly was formed on the microchannels, mimicking the multilayered thylakoids and increasing the RuBisCO loading amount in the PMRs. The (PEI/RuBisCO)*_n_*‐PMRs (where *n* denotes the number of layers) were ready for use after rinsed by reaction buffer (100 µL) at a flow rate of 2.5 µL min^−1^ to remove the weakly bounded PEI/RuBisCO assemblies. Rinse procedure was only applied after all layers have been coated.

### Confirmation of RuBisCO Immobilization

To confirm the success of RuBisCO immobilization in the PMRs by LBL assembly, the inner surfaces of the PMRs and (PEI/RuBisCO)*_n_*‐PMRs were characterized by FESEM (Tescan MAIA3), AFM (Asylum Research MFP‐3D Infinity), ATR‐FTIR (Thermo Scientific Nicolet IS50 equipped with ZnSe crystal), CD (JASCO J‐1500), and CLSM (Zeiss LSM 800 Upright Confocal Microscope, Oberkochen, Germany).

### AFM Measurements

The thickness of dried (PEI/RuBisCO)*_n_* assembly on PDMS films was measured with a scanning probe microscope (Asylum Research MFP‐3D Infinity) at tapping mode. Scratch lines were made by surgical blades to uncover the PDMS substrate and measure the thickness of the bilayers. The samples were scanned in air over an appropriate area, which consists of (PEI/RuBisCO)*_n_* assembly and exposed PDMS substrate at a scan rate of 0.5 Hz. The bilayer thicknesses were calculated by the average height difference of the line drawn at different positions including both the PEI/RuBisCO assembly and the PDMS substrate from the collected images.

### ATR‐FTIR Measurement

To obtain the ATR‐FTIR spectra, PEI/RuBisCO was deposited on thin PDMS films alternatively and then dried in the air at room temperature before the observation. For free RuBisCO, a standard KBr disk was prepared. The spectra were collected over 650–4000 cm^−1^ at the resolution of 4 cm^−1^ for 32 scans.

### Fluorescent Experiments

For the fluorescent analysis, (PEI/RuBisCO)*_n_*‐PMRs were first incubated at room temperature with 1% (w/v) BSA in PBS buffer for 30 min and then washed by PBS buffer. Then primary anti‐RuBisCO (0.5 µg mL^−1^) in PBS buffer was injected into the (PEI/RuBisCO)*_n_*‐PMRs and incubated at room temperature for 1 hour. After washing the weakly bonded antibodies by PBS at 2.5 µL min^−1^ for 20 min, AF488 GTXCH (5 µg mL^−1^) was injected into the channel and incubated at room temperature for another 1 h. Afterwards, the reactor was thoroughly rinsed by PBS buffer at 2.5 µL min^−1^ for 20 min and then observed under the fluorescence microscopy. The fluorescent images for each (PEI/RuBisCO)*_n_*‐PMRs were synthesized from 12 images that were vertically taken every 3.45 µm by focusing on the channel of the reactors. The fluorescent images were then used to plot the fluorescent intensity by ImageJ and calculated the fluorescent intensity difference between the microchannel and the microchannel wall.

### CD Measurement

PDMS thin film, RuBisCO solution, and (PEI/RuBisCO)*_n_* assembly deposited on thin PDMS films were prepared to obtain CD spectra. All the CD spectra were collected by a JASCO J‐1500 spectrophotometer at room temperature. The spectra of the solution samples were recorded over a wavelength range of 260–200 nm using a cuvette of 1‐mm pathlength at a scan speed of 50 nm min^−1^ with the data integration time of 8s, a data pitch of 0.2 nm, and a bandwidth of 1 nm. Three accumulations were carried out per data point. The concentration of RuBisCO solution was about 0.25 mg mL^−1^ in the reaction buffer. For the measurements of (PEI/RuBisCO)*_n_* on PDMS films, PEI solution (200 µL, 10 mg mL^−1^) and RuBisCO solution (0.25 mg mL^−1^) were cast onto a PDMS thin film for 1 h at room temperature alternately for different number of bilayers. The CD spectra were recorded after the films were dried at room temperature. The parameters for recording the thin films were the same as the solution except for an unidentified film thickness (pathlength). CD spectra for PDMS thin film was also recorded as the control. All the data were presented as ellipticities (*θ*, mdeg) in Figure S4 (Supporting Information).

### Determination of Protein‐Loading Amount

The rinsed‐out RuBisCO solution was collected, and the protein‐loading amount was determined by calculating the difference in protein concentration between the initially injected solution and the rinsed‐out RuBisCO solution. The protein concentration was quantified by the Bradford method^[^ ^29^ ^]^ using the Quick Start Bradford Protein Assay kit (Bio‐Rad Pacific Ltd.) and measuring the absorbance at 595 nm using a microplate reader (Thermo Scientific Varioskan LUX Multimode Microplate Reader). BSA solutions at different concentrations (0.125−1 mg mL^−1^ and 1.25−25 µg mL^−1^) were selected as standards to plot the calibration curve (Figure S3, Supporting Information). The protein‐loading amount using the immobilization method was calculated as

(1) $$\mathrm{protein}-\mathrm{loading}\;\mathrm{amount}\;\left(\mu g\right)=C_{0}V_{i}-C_{1}V_{w}$$

where *C*_0_ is the protein concentration of the injected RuBisCO solution (µg µL^−1^), *C*_1_ is the protein concentration of the rinsed‐out RuBisCO solution (µg µL^−1^), *V_i_* is the volume of the injected RuBisCO solution (µL), and *V_w_* is the volume of the washed solution collected from the outlet of the reactor (µL). The protein‐loading amounts on different (PEI/RuBisCO)*_n_*‐PMRs were recorded to find the optimal fabrication conditions.

### Residence Time and Operation Time Determination in (PEI/RuBisCO)_n_‐PMRs

The residence time *t_r_* is regarded as the residence time of the reaction mixture flowing through the (PEI/RuBisCO)*_n_*‐PMRs, which is calculated by the equation of

(2) $$t_{r}=\frac{V_{r}}{Q}$$

where *V_r_* is the volume of the (PEI/RuBisCO)*_n_*‐PMRs and *Q* is the flow rate of the injected RuBP solution controlled by the syringe pump. In this work, the volume of RI‐PMR is 7 µL, the corresponding residence time is 0.25 min, 0.5 min, 1 min, 2 min, 3.5 min, 5 min for the flow rates of 28 µL min^−1^, 14 µL min^−1^, 7 µL min^−1^, 3.5 µL min^−1^, 2 µL min^−1^, 1.4 µL min^−1^, respectively.

The operation time *t_o_* is regarded as the total operation time to collect a specific volume of the production solutions from the outlet of the (PEI/RuBisCO)*_n_*‐PMRs, which is calculated by the equation of

(3) $$t_{o}=\frac{V_{c}}{Q}$$

where *V_c_* is the volume of the collected production solutions from the outlet of the (PEI/RuBisCO)*_n_*‐PMRs and *Q* is the flow rate of the injected RuBP solution controlled by the syringe pump. In this work, when the flow rate is 7 µL min^−1^, the corresponding operation time is 30 min, 120 min, 240 min for collecting 210 µL, 840 µL, and 1680 µL respectively from one (PEI/RuBisCO)*_n_*‐PMR. If the reactor is scaled out to six parallel reactors, then the corresponding operation time will be 5 min, 20 min, 40 min for collecting 210 µL, 840 µL, and 1680 µL respectively from the six (PEI/RuBisCO)*_n_*‐PMRs.

### Assay of RuBisCO Activity

The activities of the immobilized RuBisCO were determined using a signal amplification assay adapted from the previously reported method to evaluate the amount of produced 3‐PGA.^[^ ^8^, ^30^ ^]^ Generally, reactant mixture (66 mm HCO_3_^−^ and 0.5 mm RuBP in the reaction buffer) was passed through the (PEI/RuBisCO)*_n_*‐PMRs at a flow rate of 7 µL min^−1^ (here, the residence time was 1 min). Then, the production solution (containing RuBisCO, RuBP, HCO_3_^−^ and 3‐PGA in the reaction buffer) was collected from the outlet of the reactors for further assay. The production solutions (20 µL) were added with the assay mixture (80 µL, the final concentrations were 5 unit mL^−1^ PGK, 0.5 unit mL^−1^ GAPDH, 0.5 unit mL^−1^ TPI, 0.5 unit mL^−1^ G3PDH, 1 unit mL^−1^ G3POX, 1000 unit mL^−1^ catalase, 0.5 mm ATP, 2 mm NADH, 1.5 mm MgCl_2_, and 100 mm Tricine/KOH pH 8.0). The reaction was immediately and continuously monitored by measuring the absorbance change at 340 nm by a UV‐Visible spectrometer. During the reaction, the product 3‐PGA was first converted to dihydroxyacetone‐phosphate (DAP) with PGK, GAPDH, ATP and NADH. Catalase was also added here to prevent the inhibition of GAPDH. Then, DAP was transformed into the cycle of mutual conversion with glycerol‐3 phosphate (G3P). It could be monitored as the cumulative oxidation of NADH, whose amount was much larger than the original amount of 3‐PGA, therefore providing strong amplification of signal for 3‐PGA monitoring (Figure S6a, Supporting Information). The signal of production solutions could be converted to the specific amount of 3‐PGA using a standard curve (orange dashed line in Figure S6b, Supporting Information) generated by adding different amounts of standard 3‐PGA into the assay mixture (blue squares in Figure S6b, Supporting Information). The RuBisCO activity was accordingly defined as the rate of 3‐PGA production (µmol g^−1^ RuBisCO min^−1^). The optimal concentration of RuBisCO and the optimal number of layers *n* for (PEI/RuBisCO)*_n_*‐PMRs fabrication in the following characterization and 3‐PGA production investigation experiments were determined based on the preparation of (PEI/RuBisCO)*_n_*‐PMRs that exhibited the highest immobilized RuBisCO activity.

### SDS‐PAGE Analysis

SDS‐PAGE analysis was performed by first adding the freshly prepared RuBisCO in the reaction buffer at the concentration of 5 µg µL^−1^ to 4× Laemmli Sample Buffer (Bio‐Rad Pacific Limited) at the ratio of 3:1 and then incubating at 95 °C for 10 min. Next, different amounts of the mixtures (2 µL,4 µL,8 µL, and 16 µL) were loaded to 10% SDS‐PAGE gel. Blank sample of the reaction buffer without RuBisCO (20 µL) was loaded to the first lane as the control. Finally, the stained and washed SDS‐PAGE gel was scanned by iBright FL1500 Imaging System for further analysis.

### Kinetic Study of the (PEI/RuBisCO)n‐PMRs with Optimal Fabrication Conditions

Kinetic parameters are important indicators for evaluating the enzyme immobilization method. Here, different concentrations of RuBP and 66 mm HCO_3_^−^ in reaction buffer were flowed through the (PEI/RuBisCO)*_n_*‐PMRs with optimal fabrication conditions for RuBisCO activity evaluation. The RuBisCO activities under different concentrations of RuBP were fit to a Michaelis–Menten‐type model using hyperbolic regression by GraphPad Prism 7 to derive the maximal reaction rate (*V*_max_) and the Michaelis‒Menten constant (*K*_m_) parameters of immobilized RuBisCO. The turnover number (*k*_cat_) of RuBisCO active site was calculated as *k*_cat_ = 550000  *V*_m_/8 *m*_RBC_ , where 550000 is the molecular weight assumed to be invariant among plant species, 8 is the number of active sites for holoenzyme of RuBisCO, and *m*_RBC_ is the RuBisCO concentration.^[^ ^31^ ^]^

### Feasibility of 3‐PGA Production with the (PEI/RuBisCO)n‐PMRs with Optimal Fabrication Conditions

The feasibility of producing 3‐PGA using the (PEI/RuBisCO)*_n_*‐PMRs with optimal fabrication conditions was examined by injecting 0.5 mm RuBP and 66 mM HCO_3_^−^ in the reaction buffer through the as‐prepared (PEI/RuBisCO)*_n_*‐PMRs using a syringe pump. The production solution was collected from the outlet, and the amount of 3‐PGA produced (nmol) was determined with the amplification signal assay. The injection flow rate was adjusted from 7 to 0.35 µL min^−1^ to increase the residence time from 1 to 20 min. Then, the production feasibility was evaluated by checking whether 3‐PGA production increased with increasing residence time. Control experiments were also conducted at the same flow rates for control PMRs in which the same amount of BSA was immobilized under the same conditions (BI‐PMRs).

### Thermal Stability and Storage Stability of the (PEI/RuBisCO)n‐PMRs with Optimal Fabrication Conditions

To test the thermal stability of the (PEI/RuBisCO)*_n_*‐PMRs, several prepared (PEI/RuBisCO)*_n_*‐PMRs with optimal fabrication conditions were first incubated in an oven at different temperatures (every 10 °C from 20 to 70 °C) for 10 min, and then the RuBisCO activities of each (PEI/RuBisCO)*_n_*‐PMR were examined. The (PEI/RuBisCO)*_n_*‐PMRs were also incubated at 50 °C for up to 60 min (every 10 min from 0 to 60 min) to examine their stability at elevated temperature for a prolonged time. The highest RuBisCO activity (*A*_ht_) was defined as 100%, and the relative RuBisCO activities at different incubation temperatures and incubation times were calculated as a percentage of *A*_ht_.

To test the storage stability of the (PEI/RuBisCO)*_n_*‐PMRs, several prepared (PEI/RuBisCO)*_n_*‐PMRs with optimal fabrication conditions were incubated at 4 °C for different numbers of days (0, 1, 3, 5, 10, and 15 days). Then, their immobilized RuBisCO activities were measured. The highest RuBisCO activity (*A*_hs_) was normalized as 100%, and the relative RuBisCO activities after different storage days were calculated as a percentage of *A*_hs_.

### Reusability of the (PEI/RuBisCO)n‐PMRs with Optimal Fabrication Conditions

The reusability of the (PEI/RuBisCO)*_n_*‐PMRs was determined by repeatedly carrying out the 3‐PGA production reaction with one reactor for several cycles. Here, one cycle refers to a test in which reactant mixture solution (21 µL, the minimum volume needed for the activity assay) was flowed through the (PEI/RuBisCO)*_n_*‐PMR for 3‐PGA production. The RuBisCO activity was measured from the collected product solution for each cycle of reuse. The relative RuBisCO activities were calculated as the percentage of the RuBisCO activity in the first cycle. Post‐immobilization leakage rates were determined by the slope of the linearly fitted line of the relative activities retained after each cycle of reuse.

### Continuous 3‐PGA Production with Scaled‐Out (PEI/RuBisCO)n‐PMRs with Optimal Fabrication Conditions

To examine the continuous 3‐PGA production ability of the (PEI/RuBisCO)*_n_*‐PMR system at the laboratory scale, we injected a larger volume of reactant mixture (i.e., increased the total amount injected from 21 to 1680 µL) into the PMRs while maintaining the flow rate of 7 µL min^−1^. The potential for large‐scale synthesis with (PEI/RuBisCO)*_n_*‐PMRs was evaluated by scaling out from one reactor to six parallel reactors as a proof of concept. The reactant mixture was simultaneously injected into the parallel reactors at the same flow rate (7 µL min^−1^), and then the collected product solutions from the outlets of the six parallel reactors were combined. The amounts of 3‐PGA production by one reactor and six parallel reactors were carefully compared as a function of total operation time.

### Statistical Analysis

Data are presented as mean ± S.D. The sample size is 3 (*n* = 3). One experiment was repeated three times or more in the case of the same experimental materials, experimental conditions and experimental method and representative results were presented in the figures. Calculations were performed with OriginPro 8.0, Microsoft 365, ImageJ, and GraphPad Prism 7.

## Conflict of Interest

The authors declare no conflict of interest.

## Supporting information

Supporting Information

## Data Availability

The data that support the findings of this study are available from the corresponding authors upon reasonable request.
