# Supplementary material for: Bio‐Inspired Microreactors Continuously Synthesize Glucose Precursor from CO2 with an Energy Conversion Efficiency 3.3 Times of Rice
Source: Adv Sci (Weinh). 2023 Dec 3;11(6):2305629. doi: 10.1002/advs.202305629 (PMC10853710; doi:10.1002/advs.202305629)
Supplement: Supplementary file 1 — Supporting Information [file ADVS-11-2305629-s001.pdf]

ADVANCED SCIENCE
Open Access

Supporting Information

for *Adv. Sci.*, DOI 10.1002/advs.202305629

Bio-Inspired Microreactors Continuously Synthesize Glucose Precursor from CO_2 with an Energy Conversion Efficiency 3.3 Times of Rice

Yujiao Zhu, Fengjia Xie, Tommy Ching Kit Wun, Kecheng Li, Huan Lin, Chi Chung Tsoi, Huaping Jia, Yao Chai, Qian Zhao, Benedict Tsz-woon Lo, Shao-Yuan Leu, Yanwei Jia, Kangning Ren and Xuming Zhang**

Supporting Information

Bio-Inspired Microreactors Continuously Synthesize Glucose Precursor from CO_2 with an Energy Conversion Efficiency 3.3 Times of Rice

Yujiao Zhu, Fengjia Xie, Tommy Ching Kit Wun, Kecheng Li, Huan Lin, Chi Chung Tsoi, Huaping Jia, Yao Chai, Qian Zhao, Benedict Tsz-woon Lo, Shao-Yuan Leu, Yanwei Jia, Kangning Ren, and Xuming Zhang**

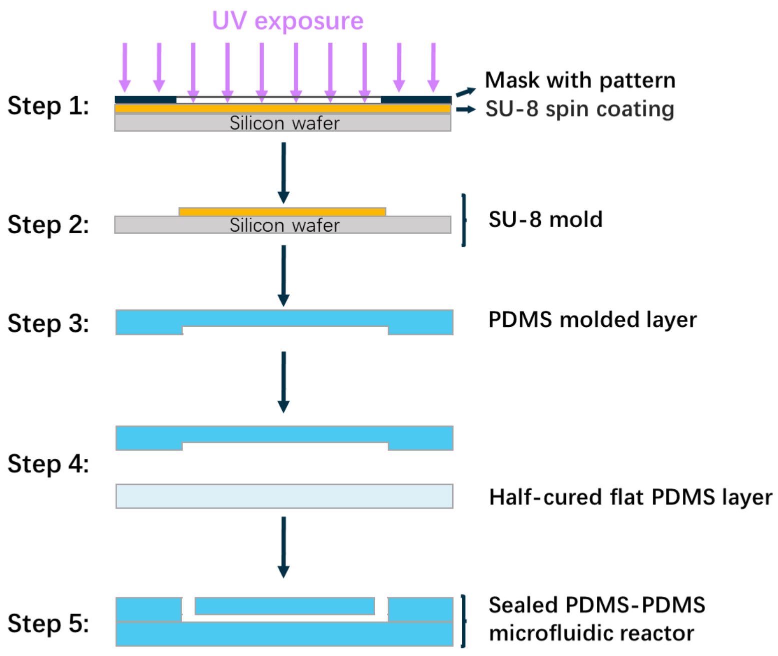

Figure S1. Process flow of the fabrication of PMRs by the soft photolithography, including photopatterning, PDMS molding and flat layer fabrication and microfluidic chip sealing.

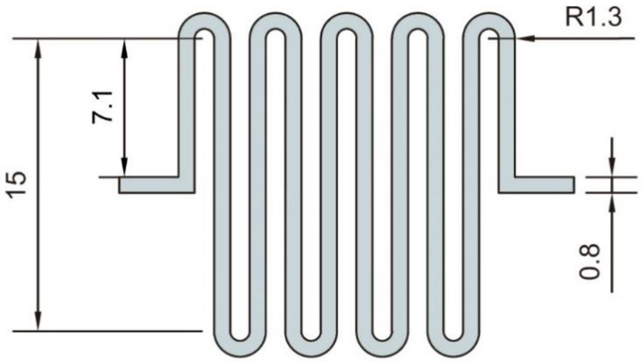

Figure S2. Detailed dimensions of the microchannels as shown by the gray-blue area. The unit is millimeter. The height of the microchannel is 40 μm.

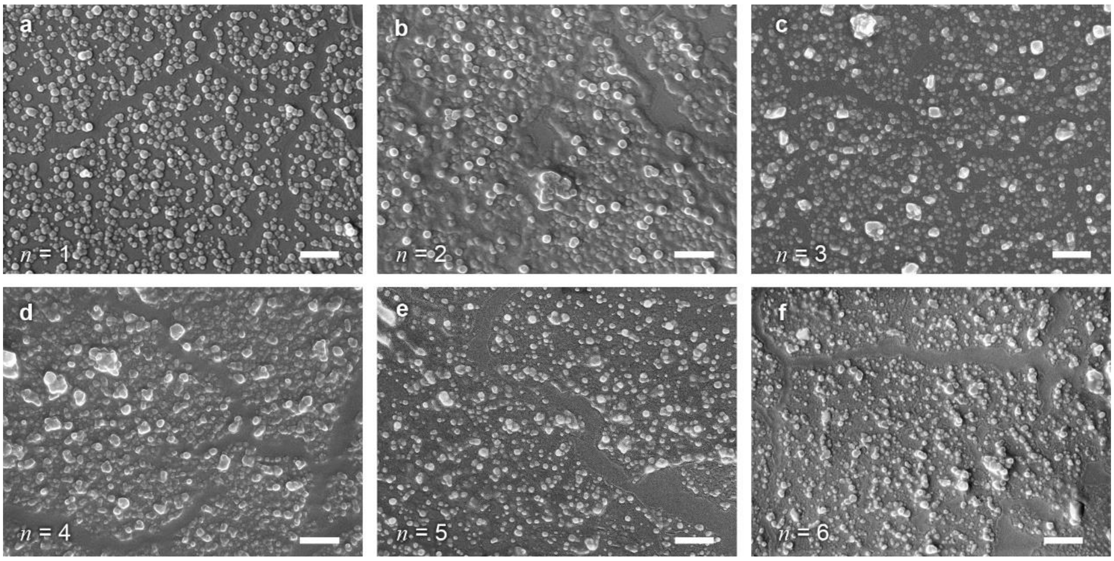

Figure S3. FESEM images of the microchannels' inner surfaces of (PEI/RuBisCO)$_n$-PMRs. (a) (PEI/RuBisCO)$_1$-PMRs, (b) (PEI/RuBisCO)$_2$-PMRs, (c) (PEI/RuBisCO)$_3$-PMRs, (d) (PEI/RuBisCO)$_4$-PMRs, (e) (PEI/RuBisCO)$_5$-PMRs, (f) (PEI/RuBisCO)$_6$-PMRs. The number of the bilayers, *n*, is indicated at the lower left of each image. The scale bars are 1 μm.

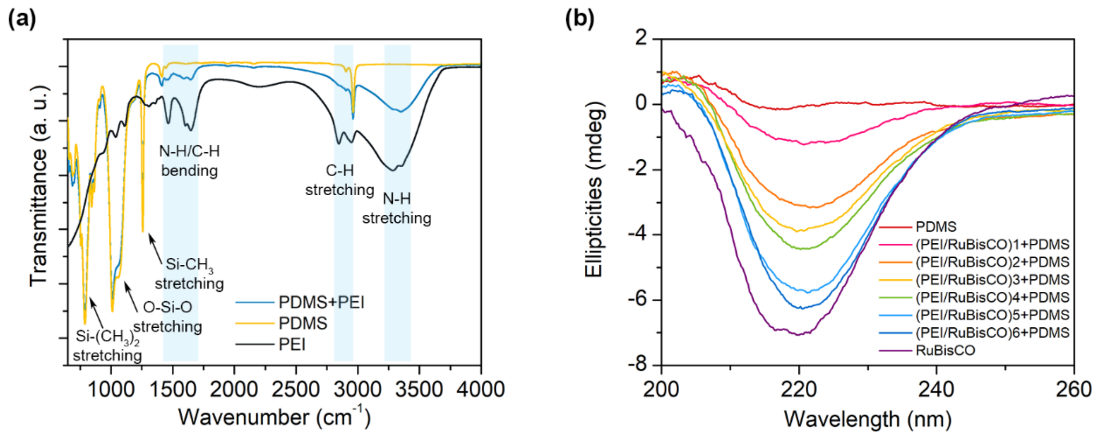

Figure S4. (a) ATR-FTIR spectra of PDMS film (yellow line), PEI solution (dark line), and PEI deposited on PDMS film (blue line). PDMS exhibit characteristic FTIR peaks at 789-796 cm^{-1} ($-CH_3$ rocking and Si-C stretching in $Si-CH_3$), 1020-1074 cm^{-1} (O-Si-O stretching), 1260-1259 cm^{-1} (CH_3 deformation in $Si-CH_3$), 2950-2960 cm^{-1} (asymmetric CH_3 stretching in $Si-CH_3$). After PEI deposition, new peaks appear at around 3200-3500 cm^{-1} corresponding to the N-H stretching vibration of amine groups in PEI, around 2800-3000 cm^{-1} corresponding to the C-H stretching vibration of methylene groups in PEI and around 1400-1600 cm^{-1} corresponding to the N-H/C-H bending. (b) CD spectra of PDMS film (red line), RuBisCO solution (purple line), and (PEI/RuBisCO)$_n$ assembly deposited on PDMS films. The spectrum of the PDMS thin film shows no peak (red line). But the RuBisCO solution has the peak at ~220 nm (purple line), indicating the secondary structure of RuBisCO[1]. The spectra of the (PEI/RuBisCO)$_n$ assembly deposited on the PDMS thin film also present the similar negative peak at ~ 220 nm, which confirms the immobilization of RuBisCO on PDMS. It is noticed that the peak intensity increases with the increasing number of the PEI/RuBisCO bilayer, suggesting the increasing amount of the immobilized RuBisCO on PDMS film, which is consistent with the ATR-FTIR spectra.

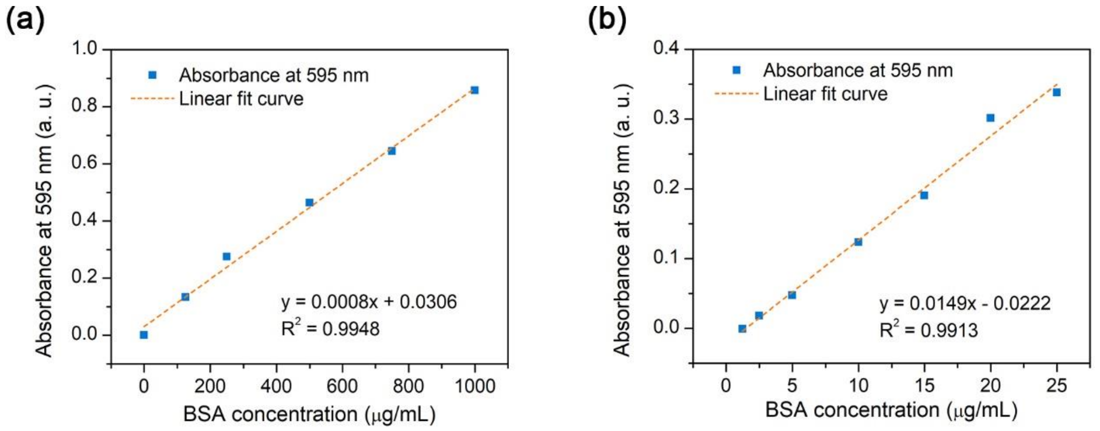

Figure S5. Calibration of protein amount determined from BSA solutions by the Bradford method. Protein amount was qualified using the Quick Start Bradford Protein Assay kit (Bio-Rad Pacific Limited.), which were determined by measuring the absorbance at the wavelength of 595 nm using a microplate reader (Thermo Scientific Varioskan LUX Multimode Microplate Reader). BSA solutions at different concentrations were selected as standards to plot the calibration curve.[2] (a) BSA at 0.125-1 mg mL^{-1} for the microplate standard assay and (b) BSA at 1.25-25 μg mL^{-1} for the microplate micro-assay.

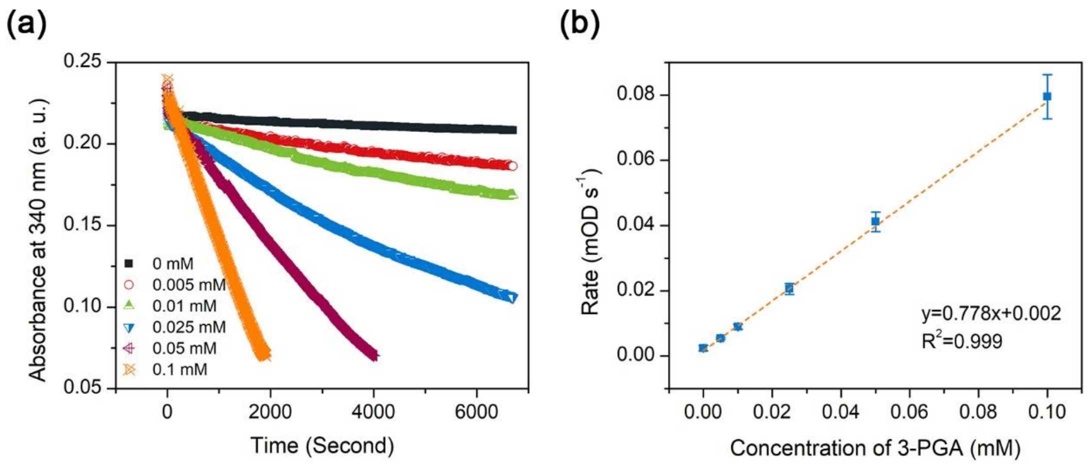

Figure S6. Calibration of standard 3-PGA amount by UV-Visible spectrometry. (a) Decrease of the absorbance at 340 nm as a function of the time for different concentrations (from 0.005 to 0.1 M) of 3-PGA dissolved in the reaction buffer. (b) Calibration line of 3-PGA in the reaction buffer by the amplification signal assay with using the microplate reader. Error bars represent the standard deviations from three independent experiments.[1]

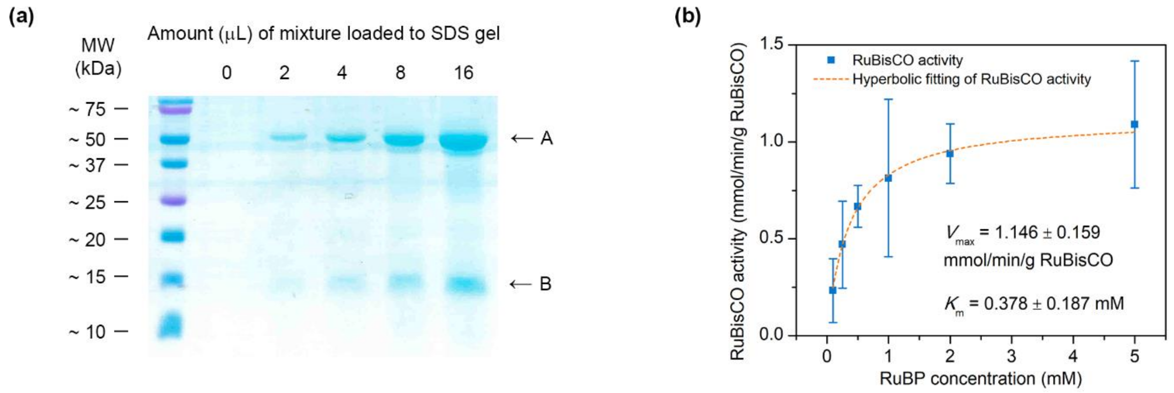

Figure S7. (a) SDS-PAGE analysis of RuBisCO in the reaction buffer for the confirmation of relative purity. The most significant protein bands are detected at ~ 53 kDa (A) and ~ 14 kDa (B) which correspond to large subunits (LSU) and small subunits (SSU) of RuBisCO, respectively. The color of protein bands tends to be darker as the loaded amount of RuBisCO increases. The relative purity of the purchased RuBisCO is estimated to be 73.4 ± 4.3%, which agrees well with the statement from Sigma that the RuBisCO (Catalogue # R8000) is partially purified; (b) Kinetics parameters of the $(PEI/RuBisCO)_1$-PMRs. Blue squares are the RuBisCO activities at different concentration of RuBP. The collected production solutions are 100 μL. The RuBP concentrations are 0.125 – 5 mM for the $(PEI/RuBisCO)_1$-PMRs reaction. The concentration of bicarbonate (HCO_3^-) in the reaction buffer is 66 mM. K_m and V_{max} values are calculated by the GraphPad Prism 7 according to the hyperbola regression fitting of Michaelis–Menten model. The hyperbola regression fitting line is shown in orange dashed curve.

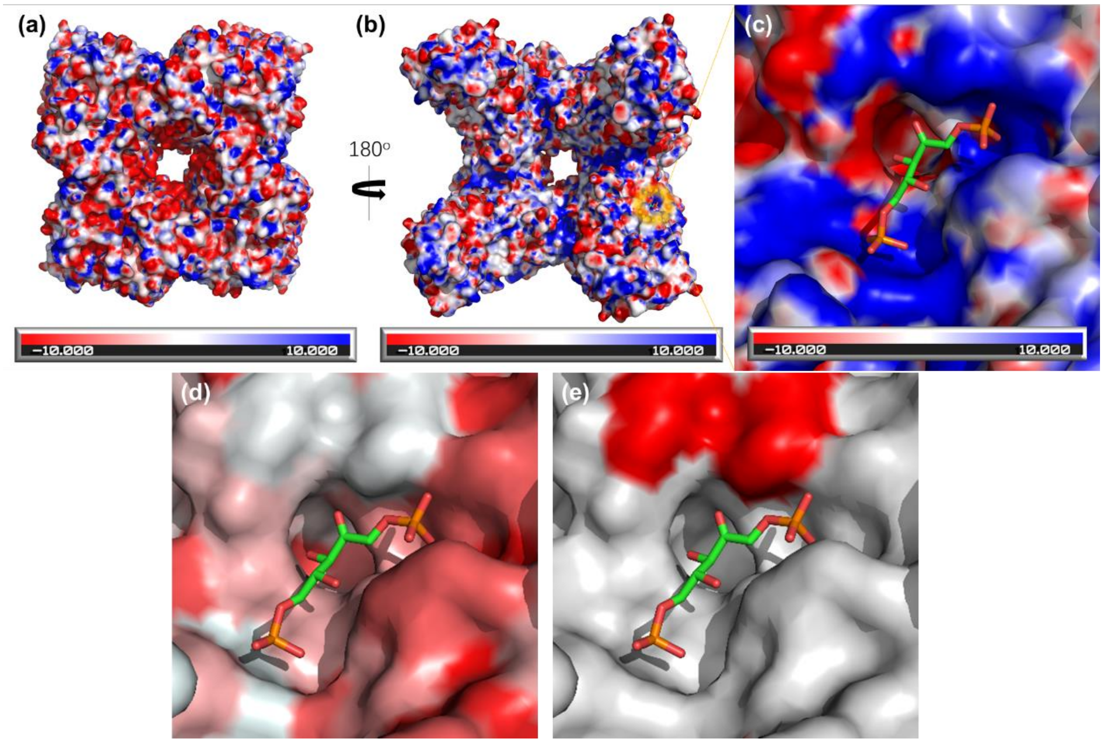

Figure S8. Visualization of the charge distribution on the surface of the RuBisCO under pH of 8.0 (positive charge - blue, negative - red, neutral – white) (a-c). (a) and (b) refer different orientations for the 3D structure of RuBisCO indicating the charge distribution; (c) shows one of the active centre of RuBisCO with RuBP; (d) shows one of the active centre of RuBisCO with RuBP visualizing the surface hydrophobicity of RuBisCO (red: hydrophobic area; white: hydrophilic area); (e) shows one of the active centre of RuBisCO with RuBP visualizing the possible covalent bonding site (red: lysine); Figures are prepared with PyMOL using the PDB coordinates 1RXO. Only half of the large and small subunits of RuBisCO structures are illustrated in the schematics.

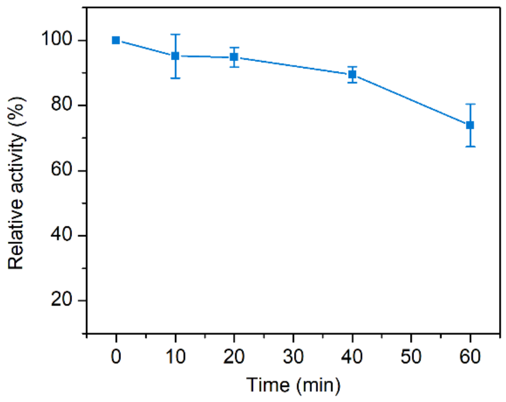

Figure S9. Relative activities of (PEI/RuBisCO)$_4$-PMRs retained after a prolonged incubation time up to 60 min at 50 ^{o}C. RuBP concentration is 0.5 mM and HCO_3^- is 66 mM. RuBP and HCO_3^- are flowed through the (PEI/RuBisCO)$_4$-PMRs continuously at the flow rate of 7 μL min^{-1}. Error bars represent the standard deviations from three independent experiments.

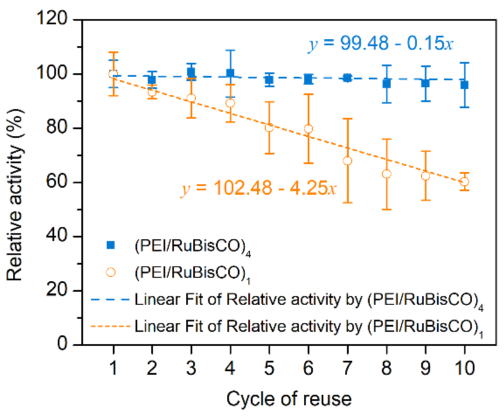

Figure S10. Comparsion of the relative activity remained after each cycle of reuse and the linear fitting lines for the $(PEI/RuBisCO)_4$-PMRs (solid blue squares and blue dash line) and the $(PEI/RuBisCO)_1$-PMRs (open orange circles and orange short dash line). About 96% of the relative activity is retained after 10 cycles of reuse for $(PEI/RuBisCO)_4$-PMRs, while only ~ 55.4% of the relative activity is retained after 10 cycles of reuse for $(PEI/RuBisCO)_1$-PMRs. The fitted post-immobilization leakage rates are 0.15% per cycle of reuse for $(PEI/RuBisCO)_4$-PMRs and 4.25% per cycle of reuse for $(PEI/RuBisCO)_1$-PMRs. RuBP (0.5 mM) and HCO_3^- (66 mM) are flowed through the $(PEI/RuBisCO)_4$-PMRs and the $(PEI/RuBisCO)_1$-PMRs continuously at the flow rate of 7 μL min^{-1}.

Table S1. Measured kinetic parameters of the immobilized RuBisCO and the free RuBisCO a).

Enzyme immobilization type	V_{max} (mmol·min^{-1}·g^{-1} RuBisCO)	K_m [RuBP] (mM)	k_{cat} (s^{-1})	k_{cat}/K_m (mM^{-1}·s^{-1})
**LBL assembly with 4 layers**	**2.494 ± 0.108**	**0.474 ± 0.069**	**2.86**	**6.03**
**LBL assembly with 1 layer**	**1.146 ± 0.159**	**0.378 ± 0.187**	**1.31**	**3.47**
Covalent immobilization b)	0.070 ± 0.003	0.070 ± 0.012	0.079	1.13
Physical adsorption c)	0.008 ± 0.001	0.090 ± 0.028	0.009	0.10
Free in solution d)	0.169 ± 0.006	0.049 ± 0.008	0.19	3.88

a) The collected production solutions are 100 μL. RuBP concentrations are 0.125 – 5 mM for the RuBisCO immobilized by LBL assembly. The concentration of bicarbonate ($HCO3^-$) in the reaction buffer is 66 mM. Km and Vmax values are the means ± s.d. of three independent experiments; b) – d) The data are collected from the previous studies.[2a, 3]

Table S2. Performance comparison of the immobilized RuBisCO and the free RuBisCO.

Enzyme immobilization type	Thermal stability [d)]	Long-term thermal stability [e)]	Storage stability [f)]	Reusability [g)]	Post-immobilization leakage rate (per cycle of reuse) [h)]
**LBL assembly**	**72%**	**74%**	**49%**	**96%**	**0.15%**
Covalent immobilization [a)]	67%	75%	43%	74%	2.94%
Physical adsorption [b)]	57%	65%	-	40%	6.61%
Free in solution [c)]	10%	47%	6%	-	-

[a) – c)] The data are collected from the previous studies using the same detection method;[2a, 3] [d)] Thermal stability is the remained relative activity after the incubation at 70 °C for 10 min; [e)] Long-term thermal stability is the remained relative activity after the incubation at 50 °C for 60 min; [f)] Storage stability is defined as the remaining relative activity after 15 days; [g)] Reusability is the remained relative activity after 10 cycles of reuse at the flow rate of 7 μL min^{-1}; [h)] Post-immobilization leakage rate is determined by the slope of the linearly fitted line of the relative activities retained after each cycle of reuse.

Supplementary References

[1] Y. Tomimatsu, J. W. Donovan, *Plant Physiol.* **1981**, *68*, 808.

[2] a) Y. Zhu, Z. Huang, Q. Chen, Q. Wu, X. Huang, P.-K. So, L. Shao, Z. Yao, Y. Jia, Z. Li, W. Yu, Y. Yang, A. Jian, S. Sang, W. Zhang, X. Zhang, *Nat. Commun.* **2019**, *10*, 4049; b) M. M. Bradford, *Anal. Biochem.* **1976**, *72*, 248.

[3] Y. Zhu, Q. Chen, C. C. Tsoi, X. Huang, A. El Abed, K. Ren, S.-Y. Leu, X. Zhang, *Catal. Sci. Technol.* **2022**.
